# Trial protocol and preliminary results for a cluster randomised trial of behavioural support versus brief advice for smoking cessation in adolescents

**DOI:** 10.1186/1756-0500-3-336

**Published:** 2010-12-14

**Authors:** Wolfgang A Markham, Christopher Bridle, Gillian Grimshaw, Alan Stanton, Paul Aveyard

**Affiliations:** 1 School of Health and Social Studies, University of Warwick, Coventry, CV4 7AL, UK; 2 Clinical Trials Unit, Warwick Medical School, University of Warwick, Coventry, CV4 7AL UK; 3 Warwick Medical School, University of Warwick, Coventry, CV4 7AL, UK; 4 Solihull Care Trust, 3, The Green, Shirley Solihull, B90 4LA UK; 5 Department of Primary Care & General Practice, University of Birmingham, Birmingham B15 2TT, UK

## Abstract

**Background:**

Many young people report they want to stop smoking and have tried to do so, but most of their quit attempts fail. For adult smokers, there is strong evidence that group behavioural support enhances quit rates. However, it is uncertain whether group behavioural support enhances abstinence in young smokers trying to quit.

**Findings:**

A cluster randomised trial for young people trying to stop smoking to compare the efficacy of a school-based 9 week intensive group behavioural support course versus a school-based 7 week brief advice only course. Participants were assessed for evidence of tobacco addiction and nicotine replacement therapy (NRT) was used if it was deemed appropriate by the therapist. Both types of course aimed to recruit approximately one hundred participants from approximately ten schools.

The primary outcome was successful quitting at 4 weeks after quit day judged according to the Russell standard. Had the trial been completed, abstinence at 6 months after quit day and the relationships between successful quit attempts and 1) psychological assessments of dependence prior to quitting 2) salivary cotinine concentration prior to quitting and 3) sociodemographic characteristics would also have been assessed. The proportion of participants who stopped smoking in each arm of the trial were compared using Chi square tests.

The trial was stopped shortly after it had started because funding to support the therapists running the stop smoking group behavioural support programme was withdrawn. Only three stop smoking courses were completed (two group support courses and one brief advice pharmacotherapy course). Seventeen participants in total entered the trial. At the end of the courses, one participant (10%) attending the group support programme had stopped smoking and no participant attending the brief advice programme had stopped smoking.

**Discussion:**

The trial was stopped so we were unable to determine whether group support helped more young people to stop smoking than brief advice. Engagement and recruitment of participants proved much more difficult than had been anticipated. Fifteen of the seventeen participants reported that quitting smoking was either pretty important or very important to them. Thus, the stop smoking success rate could, nevertheless, be considered disappointing.

**Trial registration:**

Current Controlled Trials ISRCTN25181936

## Background

The trial outlined in this study protocol aimed to compare smoking cessation success rates of QUIT Break Free intensive stop smoking group support courses for young people, with brief advice for young people. The UK charity QUIT developed the smoking cessation group support programme for young people. It is based on the Maudsley Hospital Smokers Clinic approach developed by Hajek and colleagues [1] but also draws on Bandura's social learning theory [2]. Participants in both arms of the trial were to be prescribed nicotine replacement therapy (NRT) when it was assessed as appropriate by the therapist. The trial was planned in 2005 and took place in 2007/8. It was stopped shortly after it had started because funding for the behavioural support programme was withdrawn and additional funding could not be secured. Two group support courses were completed and one brief advice course was completed before the trial was stopped. Here we outline the rationale underpinning the trial and the study protocol so other researchers working in the field of adolescent smoking cessation may benefit from our experience. Additionally, data on the recruited adolescents and their outcomes are presented and a short discussion section is included which provides up to date information on what is currently known about the efficacy of behavioural support for smoking cessation amongst adolescents.

## Rationale

The trial was initiated because adolescent smoking prevalence in the England at the time was relatively high compared with other Western European countries [3,4]. Nearly a quarter (23%) of 15 year olds in England were regular smokers in that they regularly smoked at least one cigarette per week [3]. The median consumption of 15 year old smokers was 6 cigarettes per day [3]. Most young people who quit smoking in the UK were (and still are) given little or no advice on how to do so from a health professional [5,6] indicating unmet treatment need. When smoking cessation support was offered to young people in the UK, it was commonly provided in the form of stop smoking groups together with or in the absence of nicotine replacement therapy (NRT).

The aim of the original study was to compare intensive group support with no intervention. However, shortly after the original study had begun, the Medicines and Healthcare Regulatory Authority changed the license of NRT to allow its prescription to young people aged 12 years and over. QUIT felt many young people they worked with showed evidence of tobacco addiction and decided, on the change of license, to offer NRT when it was judged appropriate. As a consequence of this and the change in licence of NRT, the intended aim of the trial was to compare group support (plus NRT when appropriate) with an unsupported quit attempt (plus NRT when appropriate). Most UK adolescents who use NRT buy it over the counter and receive minimal advice on its use. However, the QUIT therapists felt it was inappropriate to offer NRT and not supervise its use amongst young people. Therefore, the final study protocol which is reported here planned to contrast intensive group support with very brief advice on how to quit initially followed by additional supervision which focused primarily on the safe use of NRT. Both the intervention course and brief advice course were confidential and non-judgmental in keeping with all the stop smoking courses that are delivered by QUIT.

## Study protocol

### Trial Objectives

#### Aim

To examine whether intensive group support was more effective than brief advice for young people trying to stop smoking.

#### Objectives

1) To measure smoking abstinence at 4 week and 6 month follow ups in both the group support programme (intervention arm) and brief advice programme (control arm).

2) To investigate the relationships between the likelihood of successful smoking cessation and a) baseline tobacco dependence and b) socio-demographic characteristics.

## Methods

### The trial design

The trial reported here is a school based cluster randomised trial that aimed to contrast intensive group support (with or without NRT) with brief initial advice on how to quit followed by brief safety monitoring of smoking cessation pharmacotherapy.

### Eligibility criteria

In order to be eligible for enrolment into this trial, participants were required to meet all of the following inclusion criteria:

1. Attended a school that had been recruited to the trial.

2. Smoked at least one cigarette per week and was willing to attempt to stop smoking within two to four weeks.

3. Was deemed by the QUIT therapist to be suitable for smoking cessation support.

4. Was willing to attend a stop smoking course which was to be held on school premises and run during school hours, accepted that the course could either be group behavioural support or brief advice only and was willing to participate in the trial. Students who did not want to participate in the trial were allowed to attend for group behavioural support/brief advice and receive NRT if prescribed.

5. Was willing to provide her/his contact details so that she/he could be followed up. Students who did not want to give their contact details were not eligible to participate in the trial but were allowed to attend for group behavioural support/brief advice and receive NRT if prescribed.

6. Had a signed and dated a consent/assent form. Pupils provided their own consent if they were at least 16 years old or were 11-15 years old and judged by the therapist to be competent to make their own decisions regarding their health care. Pupils who were 11-15 years old who were judged by the therapist not to be competent to provide their own consent were required to obtain a signed and dated consent form from their parent/guardian. A signed and dated consent/assent form indicated the young person or her/his parent/guardian understood all aspects of both types of smoking cessation programme (group support course and brief advice course) and consented to participate in the research project (trial). Consent was obtained before pupils knew which arm of the trial they had been allocated.

### Recruitment of schools

The smoking cessation lead in the local PCT and/or the local QUIT Break Free Regional Project Managers (RPMs) selected the schools to invite into the trial and the RPMs approached and recruited schools to the trial. RPMs discussed with the head of school or a representative of the head, the aims of the trial, the two different types of stop smoking courses, advertising of the stop smoking courses and the trial, participant recruitment, consent of parents and consent/assent of participants. These discussions were supported by information sheets.

Schools were recruited if they were willing to run a stop smoking course during school time on school premises and accepted they may have been allocated a group support course or a brief advice course.

### Participant recruitment

The stop smoking courses were advertised in the school newsletter and on the school website as soon as schools agreed to become involved in the trial. The advertisements informed parents that a 7-9 week stop smoking course was to be run in the school on a weekly basis and the success of the stop smoking courses was to be evaluated by researchers. The advertisements also included the contact details of an RPM so that the wishes of parents who did not want their 11-15 year old children to become involved in the stop smoking courses and/or the trial would be upheld. Parents were encouraged to contact the RPM to discuss the stop smoking courses. During these conversations, the RPM outlined the two types of smoking cessation courses and emphasised that the type of course to be run in each school was to be decided randomly by researchers from the universities of Warwick and Birmingham.

### Training of smoking cessation therapists

The stop smoking courses for trial participants were delivered by RPMs. All the RPMs had received in-house training on running smoking cessation groups for young people. They had also attended a two day external course based on the Maudsley Hospital Smokers Clinic approach [1] which provided training on 1) how to prepare young people who want to stop smoking 2) the use of specialised forms of group-oriented activities 3) withdrawal oriented therapy which aims to help young people who smoke to overcome the effects of nicotine deprivation.

### Taster session

After a period for consideration of whether to join the course by both parents and children, the RPMs invited young smokers who wanted to give up smoking to attend a taster session (Session 1). Recruitment of participants was done through publicity, personal contacts and word of mouth. In the taster session, students were told that a weekly stop smoking course would be run in their school and that the success of the course would be evaluated by researchers from the universities of Warwick and Birmingham. Students were not told at this stage what type of stop smoking course would run in their school. The RPMs explained the nature of the two stop smoking programmes and explained the choice of course was random. The implications of the trial were also outlined. Participants were told that in later sessions they would be asked to provide their contact details and complete questionnaires. They would also be invited to participate in exhaled carbon monoxide monitoring and saliva cotinine monitoring. The ground rules of the courses and the trial were also discussed and RPMs explained to attendees at the taster session that attending the following session (Session 2) would signify commitment to stopping smoking and attending the course. RPMs answered any questions either within the group or on a confidential one-to-one basis. All pupils who attended the taster session were given an information sheet to take away with them that outlined both types of stop smoking course and the implications of the trial. The RPM also provided her contact details so that potential participants could contact her following the taster session to ask questions confidentially.

### Session 2

Participants were asked for their mobile phone numbers and email addresses to enable follow up and the RPMs provided their contact details. RPMs also asked pupils about their medical history to ensure safe prescribing of NRT and assessed for evidence of tobacco dependence. Use of NRT is standard practice in NHS smoking cessation clinics and is licensed by the Medicines and Healthcare Regulatory Authority for use in young people aged 12 years and over and endorsed by the National Institute for Health and Clinical Excellence [7]. Typically, RPMs prescribed NRT patches. The patch dose and duration of the treatment course depended upon participant's smoking behaviour. However, RPMs had a free choice of any licensed form of NRT including chewing gum and nasal spray. Participants who were given NRT used this for as long as they or their RPM deemed appropriate, but typically up to 8-12 weeks as a maximum. Once all the participants had signed the consent/assent forms during Session 2, the RPM informed them of the type of course they had all been allocated and the course started after that.

### Intervention arm - Group behavioural support programme

The group support programme took place over nine weeks including the taster session and each weekly session (30-50 minutes) provided behavioural support and advice on how to stop smoking and included NRT management. NRT was dispensed in Session 4 to commence on quit day (Session 5). The QUIT programme was not based on a single theoretical approach. Session activities and the behavioural change techniques used in the course are shown in Table 1. The identified behavioural techniques are classified according to a taxonomy of behaviour change techniques [8,9] and summarised in Additional file 1. Fidelity checks were not made.

**Table 1:** Group support course outline and behaviour change techniques 89

	Session activities	Behaviour Change Technique
**Session 1** (Taster session)	• Outline of both the group support course and the brief advice course • Outline of trial including completion of questionnaires, measurement of exhaled carbon monoxide levels and saliva cotinine levels • Discussion of confidentiality • Discussion of ground rules	• None

**Session 2**	• Introductions • Ice-breaker Game • Group Agreement • Commitment • Health check • CO reading • Set QUIT date • Forms (for researcher) • Fill in new monitoring form	• Monitoring of behaviour • Provide opportunities for social comparison • Biofeedback* • Intention formation • Specific goal setting • Agree behavioural contract

**Session 3**	• CO reading • Pre Questionnaire (Research) (HONC, FTNDQ) • Discussion around motivation for quitting • 'Weighing up Decisions' Handout • 'Smoking Diary' Handout	• Monitoring of behaviour • Provide opportunities for social comparison • Biofeedback* • Motivation interview • Provide instruction • Decision making Provide general information and information on consequences

**Session 4**	• CO Reading • Discussion around risk of smoking (where, when, feelings) • High Risk, Low risk, No risk Activity • Strategies Handout	• Monitoring of behaviour • Provide opportunities for social comparison • Biofeedback* • Behavioural information • Role play • Barrier identification • Teach to use prompts/cues

**Session 5 QUIT day**	• CO Reading • Throwing away ceremony • Buddy system: discussion around strategies • 'First Week without Smoking' Handout. • Discussion around physical affects and first week.	• Monitoring of behaviour • Provide opportunities for social comparison • Biofeedback* • Agree behavioural contract • Provide general encouragement • Buddy system* • Plan social support (emotional) • Relapse prevention • Provide instruction • Barrier identification

**Session 6**	• 1 Week post-questionnaire (MPSS) • CO Reading • Congratulations/smoking status • Difficulties? Lapse? • Use of buddies/help lines? • 'How to Survive Relapse' Handout • '2^nd ^& 3^rd ^Week without Smoking' Handout	• Monitoring of behaviour • Provide opportunities for social comparison • Biofeedback* • Provide general encouragement • Provide feedback on performance • Motivational interviewing • Provide instruction • Use follow up prompts • Review of behavioural goals • Buddy system* • Plan social support (emotional) • Relapse prevention

**Session 7**	• CO Reading • Best/Worst experiences so far • Scratch/Win Cards (Or Handout)	• Monitoring of behaviour • Provide opportunities for social comparison • Biofeedback* • Provide general encouragement • Provide feedback on performance • Motivational interviewing • Use follow up prompts • Relapse prevention

**Session 8**	• CO Reading • Smoking status/Feeling? • Show blank certificates • Do people feel healthier? Discuss • Positive Body Mapping	• Monitoring of behaviour • Provide opportunities for social comparison • Biofeedback* • Provide feedback on performance • Use follow up prompts • Motivational interviewing • Relapse prevention • Provide information on consequences

**Session 9**	• CO Reading • Smoking Status/Feeling? • Saliva Cotinine • Party and Certificates	• Monitoring of behaviour • Provide opportunities for social comparison • Biofeedback* • Provide general encouragement • Provide feedback on performance • Provide contingent reward • Use follow up prompts

*From the taxonomy outlined in 2009

### Control arm - Brief advice course

The brief advice course took place over seven weeks including the taster session. In Session 2, participants were given a brief intervention (10 tips to give up smoking) and NRT was dispensed for starting on quit day, which was Session 3. Participants were subsequently seen on a weekly individual basis by the RPM for approximately 5 minutes (Session 3 through to Session 7). During each five minute time slot, participants were offered brief one-to-one support and provided with medical management if they had decided to use NRT. Fidelity checks were not made.

### Data Collection

The RPMs collected all the data. In order to standardise data collection, all RPMs received data collection training and were provided with a data collection protocol.

### Smoking status

All participants were asked about smoking on a weekly basis and exhaled carbon monoxide was checked weekly in both the intervention and control arms, as is standard practice in smoking cessation clinics.

### Saliva cotinine monitoring

All trial participants were invited to provide a saliva sample before the quit date to corroborate their baseline questionnaire answers. Saliva cotinine samples were also taken four weeks after the quit date to validate reported abstinence (NRT permitting). Saliva samples were frozen and stored prior to analysis.

### Trial questionnaires

Baseline questionnaires were completed prior to quit day to assess symptoms of addiction to nicotine, motivation to quit and sociodemographic characteristics. Addiction to nicotine was assessed by the number of cigarettes smoked at baseline, the Hooked On Nicotine Checklist (HONC) [10,11], the Fagerstrom Test for Nicotine Dependence (FTND) [12] and the Mood and Physical Symptoms Scale (MPSS) questionnaire [13] which were all included in the baseline questionnaire. The MPSS questionnaire [13] which provides information about withdrawal symptoms after quitting smoking was also completed one week after stopping smoking. Final questionnaires that aimed to assess the perceived value of the course and satisfaction with the way the course was run were completed at the end of the course.

### Special provision for pupils who did not have well developed English reading and writing skills

The RPMs worked with students who had difficulty reading the patient information sheet and/or completing the questionnaires, without leading participants on their responses, to help them complete the tasks.

### Concomitant medication

In accordance with the National Institute for Health and Clinical Excellence guidance on smoking cessation pharmacotherapy, no other pharmacotherapy for smoking cessation was prescribed [7]. Participants continued to use their usual medication e.g. asthma inhalers.

### Safety reporting

In the event that RPMs observed moderate or severe reactions to NRT, they would have made a clinical decision on whether to stop treatment or change the dose or route of administration of NRT. The RPMs recorded inter-current illnesses on a weekly basis. Had the recorded inter-current illness contra-indicated NRT, an immediate decision about continued use of the NRT product would have been made.

### Stopping rules/Withdrawal criteria

Participants who did not attend occasional sessions were not excluded from the group support or brief advice programme or the trial. Participants who stopped using NRT or changed their NRT medication were eligible to continue with their support programme and the trial.

Participants were only classified as withdrawals if they told the RPM directly they wished to withdraw from the trial.

### Six month follow up

Six months after quit day, RPMs would have contacted each young person who attended at least Session 2 during which he/she gave his/her written consent/assent and contact details (as described above). RPMs would have telephoned or e-mailed participants to tell them she would attend their school at a particular date and time to follow up the course and assess their smoking status. At this appointment, the RPM would have confirmed smoking status, assessed exhaled carbon monoxide and taken a saliva sample for salivary cotinine measurement (NRT permitting).

### Definition of end of trial

All the smoking cessation courses were to have been completed within two years of the start date. The trial would have been completed six months and five weeks after the final group support course or six months and three weeks after the final brief advice course.

### Trial outcomes

#### Primary trial outcome

The primary outcome was quitting success at 4 weeks post quit day, as judged according to the Russell standard [14,15], meaning intention to treat and biochemically verified. This is the standard used in the NHS. Quitting smoking is defined as self-declared abstinence from smoking (not even a puff) for the previous 14 days measured at 4 weeks, confirmed by an exhaled carbon monoxide concentration of less than 8 parts per million and a saliva cotinine measurement of < 15 ng/ml (NRT permitting). The Russell standard allows a two week period of grace from quit day to accommodate lapses [16]. Those who could not be contacted for follow up (four weeks after quit day) were counted as smokers. Participants who decided to stop attending the group support course or the one-to-one brief advice course were deemed to have either continued to smoke or given up in their attempt to stop smoking.

#### Secondary trial outcomes

The secondary outcomes were 7-day point prevalence abstinence at 4 weeks and 6 months after quit day. Prolonged abstinence at six months would have been measured according to the Russell standard [14] as above.

#### Other trial outcomes (non-efficacy)

We aimed to identify the relationships between cessation and 1) psychological assessments of tobacco dependency, 2) salivary cotinine concentration prior to quitting 3) sociodemographic characteristics. We also aimed to collect information on participants' reactions to the support they received, which would have been used to develop future support programmes for young smokers.

### Trial statistical considerations

#### Power calculation

The power of the study was limited by practical constraints on the sample size. QUIT estimated they could treat approximately 200 adolescent smokers from twenty schools within a two year period, for which we were originally funded. Thus, approximately 100 participants were to be randomised to both the intervention and control arms of the intervention.

Based on QUIT's past experience, we estimated 15% of participants would have stopped smoking four weeks after quit day in the group behavioural support programme. The effect of group support was estimated to double abstinence based on evidence of effectiveness from the Cochrane review of group support in adults [17]. This would mean that 7-8% of the brief advice course participants would have stopped smoking at four weeks after quit day. A sample of around 500 would have been needed to give 80% power to exclude a difference of this magnitude. Thus, the study as planned had an unavoidable risk of a type II error.

#### Randomisation (Allocation to trial arms and treatments)

Block randomisation using blocks of four, stratified by RPM (n = 3) was used to allocate schools to the intervention or control arms. Concealed allocation was employed. Each RPM recruited the schools and telephoned the researchers as soon as the schools were recruited. The researchers, in turn, placed the school name next to the next free slot on the randomisation list which had been generated using random number tables by the researchers and telephoned the RPMs with the result of the randomisation. The RPMs had no knowledge of the allocation sequences and the researchers no knowledge or involvement with the schools.

#### Analysis

The analysis compared the proportion of participants who stopped smoking in both arms of the trial (those who received group support and those who received brief advice) using the Chi square test. However, the small number of participants limits the value of conducting this test. Rate ratios and 95% Confidence Intervals would have been calculated using standard formulae. However this was not possible as no participant stopped smoking on the brief advice course.

The possible effects of nicotine dependence and other predictors of smoking abstinence would have been examined by regression models.

### Ethics and Research Governance

The study complied with the principals of the Declaration of Helsinki (1996) and was approved by the National Research Ethics Service (07/H0718/45) and local NHS Research and Development offices. We also complied with ICH-GCP Guidelines over the reporting of adverse events, serious adverse events and suspected unexpected serious adverse reactions.

#### Monitoring and audit

The specific duties and responsibilities of the RPMs and principal investigator were outlined in a signed formal agreement between representatives of the employing organisations.

Quarterly reviews were undertaken in order to ensure young peoples' consent/assent was always sought and gained, the inclusion and exclusion criteria were exercised and the recommendations regarding NRT use among young people adhered to.

The electronic data would have been examined for consistent and logical within-person responses and these checks would have been used to ensure the data were clean. When required, the original questionnaires would have been re-examined and the electronic data amended if inaccurate.

#### Data protection and confidentiality

Data were kept in accordance with the Data Protection Act in order to protect patient confidentiality and ensure appropriate follow up. No one outside the trial team had access to either the case report forms or the database other than approved auditors from a research ethics committee, NHS Research and Development.

## Results

### Parental response to the initiative and trial

No parent from any of the three participating schools indicated they did not wish their child/children to participate in either of the stop smoking courses or the trial.

### Recruitment to the trial

All pupils who attended the stop smoking courses were deemed by their therapist to be competent to provide their own consent, were willing to enter the trial and provided their contact details in order that they could be followed up. No student who received group support or brief advice opted out of participating in the trial.

### Baseline characteristics of study participants

Two group support courses containing ten participants in total and one brief advice course containing seven participants were initiated and completed before the trial was stopped. All courses that were initiated were completed. The baseline characteristics of the participants are shown in Table 2.

**Table 2:** Baseline characteristics

	Group support (Intervention)	Brief advice course (Control)
Number of courses	2	1

Number of participants	10	7

Females (%)	5 (50)	7 (100%)

Age years (SD)	14.4 (0.7)	14.7 (0.5)

Mean number of cigarettes smoked per week (SD)	26 (17)	113 (48)

Baseline salivary cotinine (ng/ml)	85 (78)	160 (41)

Number prescribed NRT	1 (10%)	3 (43%)

Previous quit attempt	4 (40%)	6 (86%)

How important is it that you quit on this course?	Not important = 0 Slightly important = 2 (20%) Pretty important = 8 (80%) Very important = 0	Not important = 0 Slightly important = 0 Pretty important = 4 (57%) Very important = 3 (43%)

How easy or difficult would it be for you to quit smoking if you wanted to?	Very difficult = 0 Fairly difficult = 1 (10%) Fairly easy = 4 (40%) Very easy = 5 (50%) Don't know/Missing = 0	Very difficult = 1 (14.3%) Fairly difficult = 1 (14.3) Fairly easy = 2 (28.6%) Very easy = 1 (14.3%) Don't know/Missing = 2 (28.6%)

Fagerstrom Test for Nicotine Dependence	Very low dependence = 7 (70%) Low dependence = 3 (30%) High dependence = 0 Very high dependence = 0	Very low dependence = 0 Low dependence = 2 (28.6%) High dependence = 3 (42.9%) Very high dependence = 2

Mean HONC score (SD)	6 (1.9)	7.3 (3.1)

(Min score = 0, max score = 10 higher scores indicate higher dependence)		

Mean MPSS Mood score at baseline (SD) (Min score = 7, max score = 35)	13.9 (4.8)	16.6 (5.8)

The RPMs found it much more difficult to recruit participants than they had anticipated even though the three courses were run in different schools. Recruited participants tended to be members of the same friendship groups. As a consequence, most of the study participants in each school were in the same school year and had similar ages.

Relatively few participants on either course were prescribed NRT (group support n = 1; brief advice n = 3). This finding may have been related to the observation that 75% of participants who answered the relevant question thought it would be fairly or very easy to quit smoking if they wanted to. No moderate or severe reactions to NRT were observed.

Even though participants did not know which arm of the trial they had been allocated before they consented to participate in the trial, the smoking behaviour and biological assessments of dependence of participants appeared, on average, to differ markedly between the two types of smoking cessation course. Participants attending the brief advice course smoked on average more than four times the number of cigarettes per week and had saliva cotinine levels that were almost twice as high. They also appeared as expected, given the difference in the mean number of cigarettes smoked by participants, on average more dependent on cigarettes as judged by the FNTD and the HONC. Psychological assessments of dependence of attendees on the two group support courses did however, unexpectedly appear to vary according to assessment tool. Thus, participants on the group support courses appeared to have low psychological dependence on tobacco and smoking as judged by the FTND but their mean HONC score indicated they may have been more dependent on cigarettes than might have been expected given the relatively low mean number of cigarettes smoked per week.

### Outcomes

The outcomes are summarised in Table 3. Only one person sustained abstinence for 4 weeks. She was 14 years old, smoked 19 cigarettes a week, and scored low dependence on the FTND. There was no difference in the proportion of participants who stopped smoking in each arm of the trial as judged by the Chi square test. However, the small number of participants limits the value this test. Data were not available 4 weeks post quit day for one participant in each arm of the trial as they did not attend the final session. These two participants were deemed to have either continued to smoke or given up in their attempt to stop smoking. It was not possible to calculate rate ratios and 95% Confidence Intervals as no participant stopped smoking on the brief advice course.

**Table 3:** Study outcomes

	Group support course (Intervention)	Brief advice course (Control)
Number of courses	2	1

Number of participants	10	7

Mean MPSS Mood score (SD) one week after quit day (max score = 7 max score = 35)	17.9 (6.8)	20.3 (9.3)
Difference in mean MPSS Mood score after quitting and at baseline	4.0	3.7
Mean MPSS Craving score one week after quit day (SD)(Min score = 2, Max core = 12)	6.8 (2.3)	9.0 (2.5)

Russell standard 4-week abstinence *	1 (10%)	0 (0%)

* Chi square p = 0.39

## Discussion

The randomisation of schools to intervention and control arms was not completed and only three courses were run. The imbalance in smoking behaviour and biological assessments of dependence mean we cannot draw valid conclusions on the effectiveness of the intervention. The results were, nonetheless disappointing as only one person managed to quit smoking even though fifteen of the seventeen participants reported that quitting smoking was either pretty important or very important to them. Other studies have found that while young people would like to stop smoking, this is often viewed as a future aim rather than an immediate aim reflecting perhaps the more pressing nature of other issues in young people's lives [18-23]. Gnich et al. (2008) [23] also expressed disappointment at the relatively low success rates of stop smoking courses for young people in Scotland. These courses were conducted in a variety of locations and settings including schools and some of the interventions were similar to the intervention used in the trial reported here. Gnich et al. (2008) [23] proposed that if Scottish young people want and need support to stop smoking then alternative interventions might have greater impact.

The relatively low quitting success rate in this study may also have been related to the recruitment process. Even though the RPMs had prior experience of recruiting young people to smoking cessation support groups, recruitment to the trial reported here was much more difficult than had been anticipated. Recruitment of young people to stop smoking interventions in Scotland was also more difficult than expected and one pilot cessation project failed to recruit a single person within the two year evaluation period [23]. Young people's lack of interest was cited as the reason for the failure to recruit in the Scottish study and lack of interest may also have applied to the trial reported here. There are two other possible reasons for the low recruitment rate to this trial. First, young people attending school may wish to hide their smoking status and may not wish to identify themselves as smokers within a school setting. Second, although no parent indicated that they did not wish their child/children to become involved in the stop smoking courses or the trial, active parental engagement with the trial was not sought. It is possible that actively canvassing the support of parents may have resulted in improved pupil recruitment. The RPMs in the trial reported here found that participants tended to come from the same friendship groups. This may have influenced the results in two ways. First, participants may have attended the stop smoking courses to support their friends rather than to give up smoking themselves and this possibility could potentially be usefully explored in other forthcoming studies and trials. Second, recruiting friendship groups would have influenced the average age of participants, all of whom were relatively young in this trial. Gnich et al. (2008) [23] found that age was the only factor positively related to successful quitting at three month follow up.

The assumption that trial participants who dropped out have necessarily relapsed and started to smoke again may have negatively affected the reported adolescent smoking cessation quit rates in this study. Grimshaw and Stanton (2010) [6] draw attention to a variety of reasons young people are unable to attend stop smoking courses which maybe unrelated to their smoking behaviour [24-27].

Even though this trial was stopped, the preliminary results were disappointing. More encouraging findings were recorded by Grimshaw and Stanton in their Cochrane review of randomised control trials of smoking cessation interventions for young people which included nine school based trials [6]. Grimshaw and Stanton (2010) [6] concluded that complex interventions which drew on social cognitive theory and focussed on motivational enhancement and support were the most likely interventions to aid young people in their quit attempts. The intervention used in this study did, however, contain elements recommended by Grimshaw and Stanton (2010) [6].

Only one trial [28] included in the Cochrane review conducted by Grimshaw and Stanton (2010) [6] was based in the UK. The transfer of potentially effective interventions from North America to the UK although appealing may not be straightforward. Within countries, the influence of similar cognitions on adolescent smoking intentions appears relatively stable and socio-demographic characteristics such as ethnicity appear to influence intentions through these cognitions [29]. However, an investigation of the influence of country of residence on adolescent smoking intentions found that similar cognitions have very different influences on adolescent smoking intentions in different countries [30] suggesting the transfer of interventions from one country to another may be problematic.

The trial was stopped so we were unable to determine whether group support helped more young people to stop smoking than brief advice. We and others in the UK [22,23] found it much more difficult to engage and recruit participants than was anticipated. Hence, we would suggest that investigating methods for engaging and recruiting young people before setting up another randomised control trial of smoking cessation interventions for young people could be a potentially useful way forward.

## Competing interests

The authors WM, GG, AS and CB declare they have no financial or non-financial competing interests. PA has done consultancy work for Pfizer, McNeil and Xenova/Celtic, who manufacture products for smoking cessation and has no non-financial competing interests.

## Authors' contributions

PA and WM conceived of the study, participated in its design and drafted the manuscript. CG, AS, CB all participated in the design of the study and helped to draft the manuscript. All authors read and approved the final manuscript.

## Finance

The study was funded by Cancer Research UK.

## Supplementary Material

Additional file 1 **Supplemental table**. A summary of behavioural change techniques that were used in the group behavioural support programme [8,9]. Click here for file
