# Supplementary material for: Trial protocol and preliminary results for a cluster randomised trial of behavioural support versus brief advice for smoking cessation in adolescents
Source: BMC Res Notes. 2010 Dec 14;3:336. doi: 10.1186/1756-0500-3-336 (PMC3022813; doi:10.1186/1756-0500-3-336)
Supplement: Additional file 1 — Supplemental table. A summary of behavioural change techniques that were used in the group behavioural support programme [8,9]. [file 1756-0500-3-336-S1.DOCX]

**Table 2 A summary of behavioural change techniques that were used in the group behavioural support programme [8, 9]**

| **Behaviour Change Technique** |
| --- |
| Monitoring of behaviour  Provide opportunities for social comparison  Biofeedback*  Intention formation  Specific goal setting  Agree behavioural contract  Motivation interview  Provide instruction  Decision making  Provide general information and information on consequences  Behavioural information  Role play  Barrier identification  Teach to use prompts/cues  Buddy system*  Plan social support (emotional)  Relapse prevention  Use follow up prompts  Review of behavioural goals  Provide feedback on performance  Provide general encouragement  Provide contingent reward |

*From the taxonomy outlined in 2009
